# Fibroblast growth factor 19 regulates polycystic ovary syndrome progression via FGFR4-ERK-NRF2 pathway

**DOI:** 10.3389/fcell.2025.1626938

**Published:** 2025-08-29

**Authors:** Junjie Qu, Meiting Qiu, Jingyun Wang, Zhiqin Chen, Miaoxin Chen, Xiaoming Teng, Yiran Li

**Affiliations:** Department of Reproductive Medicine, Shanghai First Maternity and Infant Hospital, Tongji University School of Medicine, Shanghai, China

**Keywords:** polycystic ovary syndrome, ERK pathway, Nrf2, oxidative stress, FGF19

## Abstract

**Background:**

FGF19, an endocrine hormone, participating in ovarian function. This study investigated the roles of FGF19 in polycystic ovary syndrome (PCOS) and its associated molecular mechanisms, specifically focusing on the FGFR4-ERK-NRF2 pathway.

**Methods:**

Clinical samples were collected to determine FGF19 levels, and proteomic analysis was performed on follicular fluid. A mouse model was established to investigate the molecular pathogenesis of PCOS. Subsequently, a series of *in vitro* experiments explored the effects and mechanisms of FGF19 on PCOS with and without oxidative stress.

**Results:**

Proteomics identified 144 differentially expressed proteins enriched in pathways including VEGF, PPAR, IL-2-STAT5, mTORC1, epithelial-mesenchymal transition, bile acid metabolism, and oxidative phosphorylation. FGF19/FGF15 levels were significantly higher in PCOS patients and mice compared to controls. In PCOS mice, FGFR4, NRF2, and HO1 were upregulated, while p-ERK/ERK levels were decreased. FGF19 overexpression promoted KGN cells viability while inhibiting apoptosis, upregulating FGFR4, NRF2, HO1, BCL2, and p-ERK/ERK, and downregulating BAX. However, LY3214996 reversed the action of FGF19 overexpression in KGN cells. H2O2 treatment decreased KGN cell viability, increased apoptosis, and elevated ROS levels. NRF2 knockdown further aggravated H2O2’s effectd, whereas FGF19 overexpression countered the changes in viability, apoptosis, and ROS levels caused by H2O2. Furthermore, H2O2 stimulation upregulated BAX, NRF2, and HO1, while decreasing BCL2 and p-ERK/ERK levels; NRF2 knockdown further upregulated BAX and downregulated BCL2 and p-ERK/ERK. Conversely, FGF19 overexpression had opposite effects on NRF2 knockdown.

**Conclusion:**

FGF19 may be involved in PCOS occurrence and development through the regulation of the FGFR4-ERK-NRF2 pathway.

## 1 Introduction

Polycystic ovary syndrome (PCOS), a widespread endocrine and metabolic disorder in women of childbearing age, PCOS typically presents with excessive androgen levels, impaired ovulation, and polycystic ovarian morphology (Sadeghi et al., 2022; Zhao et al., 2023). It is estimated that one in five reproductive-aged women experiences PCOS, and struggles with its complications before menopause, with its incidence showing a progressive upward trend (Teede et al., 2023). Beyond its hallmark clinical manifestations, including hirsutism, infertility, obesity, and menstrual irregularities, PCOS significantly elevates the long-term risk of endometrial carcinoma, type 2 diabetes, and cardiovascular diseases (Patel, 2018; Guan et al., 2022; Al et al., 2021). These health complications generate severe physical and mental distress, greatly diminishing their life satisfaction and generating considerable economic pressure on households and broader communities.

Although enhanced ratios of luteinizing hormone (LH) to follicle-stimulating hormone (FSH) and increased gonadotropin-releasing hormone (GnRH) pulse frequency are recognized as underlying contributors to PCOS (Bednarska and Siejka, 2017), the precise etiology and pathophysiology remain elusive. Recently, the pathogenesis of PCOS has emerged as a focal point in reproductive medicine research, with oxidative stress recognized as a pivotal player in its initiation and progression (Singh et al., 2023). Under normal physiological conditions, a dynamic equilibrium exists between the oxidative and antioxidant systems. However, in PCOS patients, higher androgen levels and insulin resistance disrupt this balance, leading to excessive accumulation of reactive oxygen species (ROS) (Siddiqui et al., 2022). The resultant ROS overload not only inflicts damage to cellular lipids, proteins, and DNA but also dysregulates critical signaling pathways (Cheung and Vousden, 2022). These perturbations contribute to aberrant follicular development, exacerbated insulin resistance, and chronic inflammatory responses, collectively amplifying the trajectory (Kumariya et al., 2021). Therefore, deciphering the mechanistic involvement of oxidative stress in PCOS pathogenesis could revolutionize preventive strategies, diagnostic approaches, and therapeutic interventions while potentially enhancing *in vitro* fertilization (IVF) success rates.

Fibroblast growth factor 19 (FGF19, also called the FGF15 homologue in mice), a member of the fibroblast growth factor family, functions as an endocrine hormone that plays a crucial regulatory role in glucose/lipid homeostasis and bile acid metabolism (Vonderohe et al., 2023). Emerging evidence suggests that FGF19 may also participate in the regulation of ovarian function through the paracrine or endocrine pathways, demonstrating a potential association with PCOS (Zhu et al., 2023; Ramanjaneya et al., 2020). Cheng et al. (2021) showed that FGF19 levels were markedly elevated in PCOS subjects compared to healthy individuals (*P* < 0.001), and were positively correlated with the progression of diabetes in controls. Additionally, FGF19 can bind to specific receptors (e.g., FGFR4) to activate downstream signaling pathways and participates in a series of biological functions, including differentiation, metabolic homeostasis, proliferation, and oxidative stress responses, thereby playing important roles in the management of cholestatic disorders, metabolic diseases, and certain malignancies (Li X. et al., 2024; Xu et al., 2023; Xie et al., 2023). The ERK pathway is a fundamental intracellular signaling cascade that mediates essential cellular processes, including growth, differentiation, and survival (Crozet and Levayer, 2023). As the central regulator of redox homeostasis, nuclear factor erythroid 2-related factor 2 (NRF2) transcriptionally controls a network of genes encoding antioxidant enzymes (Tossetta et al., 2022). Notably, heme oxygenase 1 (HO1) is a key NRF2 target gene that exerts cytoprotective effects through its potent antioxidant and anti-inflammatory activities (O'Rourke et al., 2024). However, the precise functions and underlying mechanisms of action of FGF19 in the initiation and progression of PCOS remain unclear.

Therefore, we first unearthed the molecular pathogenesis of PCOS using proteomics analysis and then investigated the effects of FGF19 on PCOS and its associated molecular mechanisms, with an emphasis on the regulatory action of the FGFR4-ERK-NRF2 pathway. These findings provide critical insights into the pathogenesis of PCOS, offering novel theoretical foundations and potential therapeutic targets for PCOS diagnosis and treatment with significant scientific and clinical implications.

## 2 Materials and methods

### 2.1 Patients and clinical samples

From August 2021 to October 2021, 26 patients with PCOS and 26 control individuals were recruited from the Reproductive Center, Shanghai First Maternity and Infant Hospital (Shanghai, China). Blood samples were obtained from each subject to determine the levels of FGF19 using the human FGF-19 ELISA Kit (ImmunoDiagnostics Limited, Shenzhen, China) according to the manufacturer’s instructions. Subsequently, the correlation between FGF19 and PCOS-related indices was analyzed. PCOS-related indices refer to key clinical and biochemical parameters closely associated with the diagnosis and pathophysiology of PCOS, including reproductive hormones [FSH, LH, estradiol (E2), prolactin (PRL), progesterone, and testosterone] and anti-Müllerian hormone (AMH), which reflect ovarian reserve, hormonal imbalance, and follicular development characteristics in PCOS patients (Li et al., 2022; Kulkarni et al., 2023).

Additionally, 3 PCOS patients undergoing IVF treatment and three controls were recruited to collect follicular fluid samples for subsequent proteomics. The inclusion criteria were as follows: three patients with PCOS and three control individuals who underwent oocyte retrieval at our reproductive center in September 2024 were randomly selected, were under 35 years of age, and underwent first-time oocyte retrieval. Participants aged >35 years, those with a history of oocyte retrieval, and those with a history of recurrent miscarriages were excluded. The participant characteristics, including demographic and clinical features, are presented in Table 1. The study protocol was reviewed and approved by the Institutional Ethics Committee of Shanghai First Maternity and Infant Hospital, and all participants provided informed consent following the national laws and Declaration of Helsinki guidelines.

**TABLE 1:** The basic clinical information of polycystic ovary syndrome (PCOS) patients and control individuals.

Sample type	Group	No.	FSH (IU/L)	LH (IU/L)	E2 (pg/mL)	PRL (ng/mL)	P (ng/mL)	T (ng/mL)	AMH (ng/mL)
Blood	Control	1	11.08	2.89	42.18	26.1	0.56	0.18	3.20
		2	4.90	2.53	59.43	8.59	0.45	0.45	1.99
		3	5.00	2.80	30.10	11.34	0.23	0.35	3.23
		4	9.56	3.27	33.90	12.41	0.26	0.24	4.76
		5	7.59	5.07	41.61	9.75	0.81	0.26	2.51
		6	13.58	4.07	38.91	8.34	0.64	0.23	0.62
		7	8.08	13.93	66.00	15.10	0.59	0.34	6.48
		8	5.78	3.15	53.00	9.68	0.25	0.14	1.17
		9	6.07	2.67	22.33	10.57	0.57	0.21	2.24
		10	4.24	1.85	23.78	30.00	0.74	0.30	5.01
		11	1.02	3.61	82.00	15.12	0.42	0.27	3.41
		12	5.60	5.78	30.65	9.57	0.58	0.24	5.91
		13	7.59	5.07	41.61	10.87	0.81	0.26	2.51
		14	6.63	13.48	94.10	17.61	0.68	0.27	13.10
		15	8.36	4.52	29.05	7.26	0.58	0.15	3.12
		16	8.56	5.84	49.73	11.03	0.77	0.26	5.34
		17	11.69	3.10	42.64	10.95	0.43	0.22	2.18
		18	3.45	1.34	54.00	17.68	0.28	0.21	2.11
		19	9.41	4.07	57.28	11.04	1.38	0.51	1.37
		20	7.75	8.40	25.00	14.43	0.45	0.26	2.31
		21	8.68	6.33	73.38	17.46	0.63	0.25	3.03
		22	5.88	1.86	37.74	13.88	0.91	0.35	5.53
		23	9.58	9.07	66.00	13.33	0.36	0.16	3.24
		24	5.19	2.76	36.15	12.51	0.57	0.68	1.75
		25	25.95	13.09	20.81	11.39	0.89	0.47	0.02
		26	9.07	2.57	37.33	21.19	0.82	0.25	2.53
	PCOS	1	7.70	9.04	47.87	21.28	0.41	0.29	8.32
		2	6.20	9.29	45.52	8.44	0.14	0.51	10.12
		3	5.24	5.72	36.93	10.55	0.51	0.36	8.67
		4	5.96	15.32	57.34	17.14	0.53	0.56	10.99
		5	4.78	9.65	41.04	14.56	0.37	0.34	12.36
		6	5.21	0.34	21.13	18.65	0.44	0.42	4.76
		7	7.08	8.56	42.5	21.04	0.64	0.24	10.10
		8	7.58	7.35	54.2	13.31	0.42	0.20	4.47
		9	5.58	1.66	16.69	13.25	0.70	0.21	3.78
		10	2.58	0.85	61.46	12.10	0.52	0.98	24.03
		11	4.62	5.78	45.78	11.25	0.36	0.38	5.76
		12	6.63	13.48	94.10	17.61	0.68	0.27	13.10
		13	5.69	5.55	44.48	10.55	0.93	0.51	8.93
		14	5.93	4.46	43.00	7.75	0.43	0.41	2.53
		15	6.41	21.34	67.47	14.21	1.29	0.41	8.87
		16	8.67	8.59	56.34	8.89	0.38	0.46	9.97
		17	7.87	12.95	28.60	23.52	0.84	0.20	8.34
		18	6.74	17.10	68.57	10.34	0.56	0.50	7.84
		19	3.47	10.2	51.1	8.84	0.74	0.35	13.83
		20	4.98	5.78	38.90	8.64	0.58	0.29	5.94
		21	5.62	4.67	36.80	13.15	0.38	0.36	4.54
		22	4.92	11.82	37.66	14.68	0.67	0.41	22.76
		23	5.76	6.09	49.59	11.46	0.81	0.46	11.65
		24	5.63	5.44	20.77	15.41	0.37	0.24	6.07
		25	12.34	7.06	36.71	9.50	0.66	0.28	7.97
		26	7.23	7.33	62.87	20.63	0.58	0.35	15.28
Follicular fluid	Control	1	5.54	3.23	28.03	19.95	0.81	0.19	1.95
		2	3.47	5.29	12.84	9.93	0.34	0.16	2.96
		3	4.68	1.89	23.27	2.9	0.73	0.23	2.9
	PCOS	1	6.26	7.39	34.64	21.66	0.42	0.47	17
		2	4.01	13.2	25	12.3	0.455	0.34	15.04
		3	3.63	7.07	34.5	13.4	0.55	0.34	9.21

FSH, follicle-stimulating hormone; LH, luteinizing hormone; E2, estradiol; PRL, prolactin; P, progesterone; T, testosterone; AMH, anti-Müllerian hormone.

### 2.2 Protein extraction and proteomics analysis

The follicular fluid samples from each group (n = 3) were sent to Jiahua Yaorui Technology Co., Ltd. (Beijing, China) for proteomic analyses. Briefly, the follicular fluids were thawed at 4 °C, and 100 μL of follicular fluid was incubated in 100 μL of beads (1 mg/mL). After binding at 37 °C for 1 h, 200 μL of wash buffer (10 mM tris(hydroxymethyl)aminomethane [Tris], pH = 7.4; 150 mM potassium chloride; 0.05% 3-[(3-cholamidopropyl)dimethylammonio]-1-propanesulfonate [CHAPS]) was added. Based on the methods of Liu et al. (Li et al., 2022), the bead incubation, reduction, and alkylation were performed according to the methods described by Li et al. (2022). To the bead precipitate, lysis buffer (0.5% sodium deoxycholate monohydrate [SDC] (w/v), 10 mM Tris (2-carboxyethyl)phosphine hydrochloride [TCEP], 40 mM calcium acetylacetonate [CAA], 50 mM tetraethylammonium bromide [TEAB]) was add. After sonication for 10 min and centrifugation, the supernatant was transferred to a new tube and this step was repeated once. Supernatants from both steps were combined and heated at 95 °C for 10 min in a metal bath. For digestion, 0.5 μg of trypsin (Promega, United States) was used, and cultivated for 16 h at 37 °C. Thereafter, the digested peptide sample was desalted using a 96-well plate (Model PLATE NBE ATLAS 96-Well 2.5 mg) following the instructions provided with the desalting kit.

The prepared samples were used for liquid chromatograph-mass spectrometer (LC-MS) using the Vanquish Neo UHPLC system (Thermo Fisher Scientific, United States). Following dissolution in 0.1% (v/v) aqueous formic acid, peptide samples (200 μg) were injected onto a C18 pre-concentration column (Acclaim PepMap 100, 5 μm, 100Å, Thermo Fisher) for desalting and concentration, as well as then chromatographically resolved using a laboratory-prepared analytical column (17 cm in length, 75 μm in internal diameter). The temperature of sample injector and column was respectively 8 °C and 35 °C. The flow rate was 0.25 mL/min, and the injection volume was 2 μL. The mobile phase consisted of solvents A (0.1% formic acid in water) and B (80% acetonitrile/0.1% formic acid). The gradients of chromatographic separation were as follows: 6.7%–24% B for 11.3 min; 24%–36% B for 5.7 min; 36%–55% B for 1.5 min; 55%–99% B for 0.5 min; as well as 99% B for 5 min. The samples were then analyzed by high-resolution mass spectrometry (Orbitrap Astral, Thermo Scientific) using positive-ion mode electrospray ionization at 2.2 an applied potential. The MS1 scan range was 380–980 m/z, with a resolution of 240,000. The DIA MS2 isolation range was 380–980 m/z, with an isolation window width of 2 m/z, a scan time of 3 ms, an MS2 scan range of 150–2000 m/z, and a normalized collision energy of 25. The automatic gain-control (AGC) setting were 5e5.

Raw mass spectrometry data were converted to mzML format using MSConvert (v3.0.21072) and subsequently analyzed using DIA-NN software (v1.8). The human database was downloaded from Uniport _Homo_sapiens_UP000005640.fasta with 20588 entries. A library-free quantity was used for the database search. Trypsin was used for enzymatic digestion, allowing for no more than two missed cleavages and peptide lengths of less than 7 amino acids. Cysteine carbamidomethylation was among the fixed modifications, whereas methionine oxidation and N-terminal acetylation were considered variable modifications. The mass tolerance for both MS1 and MS2 was set to 20 ppm, with a false discovery rate (FDR) set at 1%, and the match between run (MBR) features was enabled. After searching the sequence database, we used Python (v3.10.4) for quantification data preprocessing, including normalization and missing-value imputation. After that, differential expressed proteins (DEPs) were identified using R software (v4.2.2), with the thresholds of fold change (FC) ≥ 2.0 or FC ≤ 0.5, and p-value <0.05. After that, the identified DEPs were subjected for functional analyses, including Kyoto Encyclopedia of Genes and Genomes (KEGG) as well as Gene Set Enrichment Analysis (GSEA).

### 2.3 Establishment of a PCOS mouse model, and sample collection

Twenty female C57BL/6 mice were procured from Sipefu Biotechnology Co., Ltd. (Beijing, China). These mice were housed in an environment where the temperature was 20 °C–25 °C as well as the humidity was 50 ± 5%, and they were exposed to a 12-h light/dark cycle. Throughout the experiments, mice had unrestricted access to food and water. Following a 3-day acclimation period, all mice were randomly assigned to the control and PCOS groups, with ten mice in each group. Mice in the control group received a daily subcutaneous injection of 0.2 mL of sesame oil at the neck nape and were fed a standard diet. However, the mice in the PCOS group were injected with dehydroepiandrosterone (DHEA, 60 mg/kg/d) in 0.2 mL sesame oil subcutaneously on the nape of the neck daily and fed a 60% high-fat diet.

Starting on experimental day 10, the estrous cycle of mice was monitored daily for 10 consecutive days. Vaginal swab smears were obtained at a fixed time each morning. An appropriate amount of 0.9% normal saline was absorbed using a Pasteur pipette and gently flushed into the vaginal orifice to collect the secretions. The samples were then transferred onto glass slides, stained using a rapid, non-toxic, modified Papanicolaou (PAP) staining kit (Jiancheng Bioengineering Institute, Nanjing, China), and observed under a light microscope to determine the estrous phase based on cellular morphology.

Prior to the experimental endpoint, the mice were a 16-h fasting period (from 5:00 p.m. on the previous day to 9:00 a.m. on the test day) with free access to water for oral glucose tolerance tests (OGTT). Briefly, fasting blood glucose levels were measured in tail vein blood samples at time point 0 (fasting baseline). Subsequently, a glucose solution (20% w/v, 100 μL/10 g) was administered by oral gavage; as well as post-administration glycemic profiles were determined at 30-min intervals up to 120 min via tail vein blood draws. Glucose tolerance was assessed by analyzing blood glucose dynamics over time.

After completion of the experiment, blood samples were collected from each mouse. After sacrifice by cervical dislocation, the ileum, liver, and ovarian tissues were collected from control and PCOS mice for further experiments.

### 2.4 Hematoxylin eosin (HE) staining

Ovarian tissues were immersed in 4% paraformaldehyde (PFA) and fixed for 24 h. Subsequently, they were dehydrated using a sequence of ethanol solutions of increasing concentrations (70%, 80%, 90%, 95%, and 100%), cleared with xylene, and finally embedded in paraffin. After slicing into 5-μm-thick sections, they were firstly stained with hematoxylin for 5 min, followed by eosin for 3 min. After mounting with neutral resin, stained slides were observed and imaged under a light microscope (Nikon Corporation, Tokyo, Japan).

### 2.5 Immunohistochemistry (IHC)

Ovarian tissue sections were baked at 60 °C for 1 h, dewaxed in xylene, and dehydrated using an ethanol gradient. After rinsing with PBS, antigen retrieval was performed using a sodium citrate-hydrochloric acid buffer. After quenched endogenous peroxidase for 10 min, samples were sealed with 5% goat serum for 1 h at 25 °C. The samples were then incubated with an anti-FGFR4 antibody (1:200, Abcam, Cambridge, UK) overnight at 4 °C, followed by incubation of HRP-conjugated anti-rabbit IgG (1:1000, Proteintech, Wuhan, China) at 37 °C for 60 min. After staining with DAB (Beyotime, Shanghai, China) and hematoxylin, and sealing with neutral resins, the images were observed and photographed under a microscope (Nikon Corporation).

### 2.6 Enzyme-linked immunosorbent assay (ELISA)

Blood samples were obtained to determine the levels of FGF15 (i.e., FGF19 in humans), FSH, and testosterone in the control and PCOS mice using the FGF15 ELISA Kit (7TM Antibodies, Jena, Germany), FSH (Rodent) ELISA Kit (Abnova, Taiwan, China), and testosterone ELISA Kit (Abcam), respectively, in accordance with the manufacturer’s protocols.

### 2.7 Cell culture and transfection

Procella (Wuhan, China) provided human ovarian granulosa cells (KGN cells), which were cultured in DMEM/F12 (Thermo Fisher Scientific, United States) supplemented with 10% fetal bovine serum (FBS, Thermo Fisher Scientific) and 1% penicillin/streptomycin (Thermo Fisher Scientific). The KGN cells were maintained in an incubator at 37 °C with 5% CO_2_.

Cells were transfected using Lipofectamine 3000 (Thermo Fisher Scientific) following the manufacturer’s recommendations. Briefly, si-NRF2-1/2/3, si-negative control (si-NC), oe-FGF19 (pcDNA3.1+-FGF19), and oe-NC (oe-NC, pcDNA3.1+) were designed, synthesized, and provided by Yanzai Biotechnology (Shanghai, China). The sequences of si-NRF2-1/2/3 were shown as follows: si-NRF2-1, GAGAAAGAATTGCCTGTAA; si-NRF2-2, GCTACGTGATGAAGATGGA; si-NRF2-3, GCCCTCACCTGCTACTTTA; and si-NC, TTCTCCGAACGTGTCACGT. A total of 2 × 10^4^ KGN cells per well were seeded in a 24-well plate and incubated overnight. The next day, the culture medium was replaced with a serum-free medium. Then, 4 μg of oe-FGF19 plasmids, 4 μg of oe-NC, 20 nM of si-NC, or 20 nM of si-NRF2 were respectively transfected to KGN using Lipofectamine 3000. Six hours post-transfection, the medium was replaced with a complete medium. Following a further 24-h incubation period, the transfection efficiency was examined by evaluating FGF19 or NRF2 expression using real-time quantitative PCR (RT-qPCR) or Western blotting.

### 2.8 Cell grouping

To investigate the role of FGF19 in PCOS, KGN cells were randomly divided into four groups: oe-NC, LY3214996 (an ERK inhibitor), oe-FGF19, and oe-FGF19+ LY3214996. KGN cells in the oe-NC and LY3214996 groups were first transfected with oe-NC plasmid and then treated with DMSO and 10 nM LY3214996. Cells in the oe-FGF19 and oe-FGF19+ LY3214996 groups were first transfected with oe-FGF19 plasmid and then treated with DMSO and 10 nM LY3214996.

Furthermore, we explored the interaction between FGF19 and NRF2 in high-oxidative stress-induced PCOS progression. KGN cells were randomly assigned to PBS, H_2_O_2_, H_2_O_2_+si-NC, H_2_O_2_+si-NRF2, H_2_O_2_+oe-FGF19, and H_2_O_2_+si-NRF2+oe-FGF19 groups. Apart from the PBS-treated cells, KGN cells in the rest groups were firstly administrated with H_2_O_2_ (100 μM) for 4 h, and then transfected with corresponding si-NC, si-NRF2, and oe-FGF19.

### 2.9 Cell viability and apoptosis assays

KGN cells under different treatments were harvested for determination of cell viability and apoptosis using a cell counting kit-8 (CCK-8, BBI Life Sciences) and an Annexin V-FITC/PI cell apoptosis assay kit (YEASEN, Shanghai, China), respectively. For cell viability, the harvested cells were added with 10 μL CCK-8 reagent. After 2 h of incubation, the absorbance was measured at 450 nm using a microplate reader (Biotek, Vermont, United States).

For cell apoptosis, the cells after centrifugation (1,000 rpm, 5 min) were resuspended in 195 μL Annexin V-FITC binding buffer, stained with 5 μL Annexin V-FITC and 5 μL propidium iodide (PI), and incubated for 20 min at 25 °C in the dark. Flow cytometry (Becton Dickinson) was used for imaging, and apoptosis rates were quantified using the CellQuest software.

### 2.10 Determination of reactive oxygen species (ROS) levels

To examine ROS production, KGN cells under different treatments were subjected to flow cytometry using a commercial ROS assay kit (Beijing Biao Leibo Technology Co. Ltd., Beijing, China). The cells were pelleted (1,000 rpm, 5 min), stained with 10 μM DCFH-DA in PBS for 30 min at 37 °C. After washing, the cells were resuspended in complete medium and analyzed using flow cytometry.

### 2.11 Real-time quantitative PCR (RT-qPCR)

Total RNA was extracted from cells or tissues under various treatments using the TRIzol reagent (Invitrogen, United States). Subsequently, the RNA was reverse-transcribed into cDNA using a first-strand synthesis kit (TransGen Biotech, China), following the manufacturer’s instructions. After that, the synthetic cDNA was applied for qPCR, with the reaction system including 10 μL 2 × AceQTM Universal SYBR qPCR Master Mix (Vazyme China), 8 μL DNA, 0.5 μL forward/reverse primers (10 μM), as well as sterile water (up to 20 μL). The mRNA expression of these genes was calculated by the 2^−ΔΔCT^ method with glyceraldehyde-3-phosphate dehydrogenase (GAPDH) as the housekeeping gene. The sequences of all the primers are listed in Table 2.

**TABLE 2:** Primer sequences.

Primer	Sequences (5′-3′)
m-FGF15-F	GGTCCCTATGTCTCCAACTGC
m-FGF15-R	CTTGATGGCAATCGTCTTCAGA
m-FXR-F	GCTTGATGTGCTACAAAAGCTG
m-FXR-R	CGTGGTGATGGTTGAATGTCC
m-GAPDH-F	AGGTCGGTGTGAACGGATTTG
m-GAPDH-R	GGGGTCGTTGATGGCAACA
m-CYP7a1-F	GGCTAGCATAGCCAACTTGC
m-CYP7a1-R	CAGACAAAGCACTTGCCCTTC
m-CYP8b1-F	ACTTGACTCATCCTGGAGGGC
m-CYP8b1-R	TCCATTGAGCAACATCCCTGG
m-FGFR4-F	CCTTCCACGGGGAGAATCG
m-FGFR4-R	CTCCACAAGGCATGTGTATGT
h-Nrf2-F	TTCCCGGTCACATCGAGAG
h-Nrf2-R	TCCTGTTGCATACCGTCTAAATC
h-GAPDH-F	ACAACTTTGGTATCGTGGAAGG
h-GAPDH-R	GCCATCACGCCACAGTTTC

### 2.12 Western blot

Cells subjected to different treatments, along with tissue samples, were lysed in RIPA buffer for total protein extraction. The extracted protein was quantified using a BCA assay kit (Beyotime, Shanghai, China). Following this step, protein samples (20 μg per lane) were electrophoresed on 10% SDS-polyacrylamide gels and subsequently transferred to PVDF membranes. After blocking with 5% skim milk at 37 °C for 1 h, the membranes were incubated with primary antibodies against NRF2 (cat. no. ab89443, 1: 1000, Abcam), FGFR4 (cat. no. 11098-1-AP; 1:1000; Proteintech) and FLAG (cat. no. 80010-1-RR, 1:10000, Proteintech); ERK (cat. no. 11257-1-AP; 1:1000; Proteintech) and BAX (cat. no. 50599-2-Ig; 1:1000; Proteintech) and p-ERK (cat. no. #4695, 1:1000; Cell Signaling Technology) and BCL2 (cat. no. A0208, 1:1000; Abcam), HO1 (cat. no. 82206, 1:1000, Cell Signaling Technology) and GAPDH (cat. no. #5174, 1:20000, Cell Signaling Technology) overnight at 4 °C. After washing, the membranes were incubated with goat anti-mouse/rabbit IgG (H + L)-HRP (cat. no. #7076 or #7074, 1:5000, Cell Signaling Technology) at 37 °C for 2 h. Finally, the immunoreactive bands were detected using a Millipore enhanced chemiluminescence (ECL) detection kit (Beyotime).

### 2.13 Statistical analysis

Results are reported as mean ± SD of triplicate experiments. Statistical significance was evaluated using SPSS (IBM, United States) and figures were created using GraphPad Prism 9 (GraphPad Software, United States). For two-group comparisons, an unpaired t-test was used, whereas a one-way ANOVA was used for multi-group analyses. Statistical significance was set at *P* < 0.05.

## 3 Results

### 3.1 Higher FGF19 expression in PCOS

**FIGURE 1:**
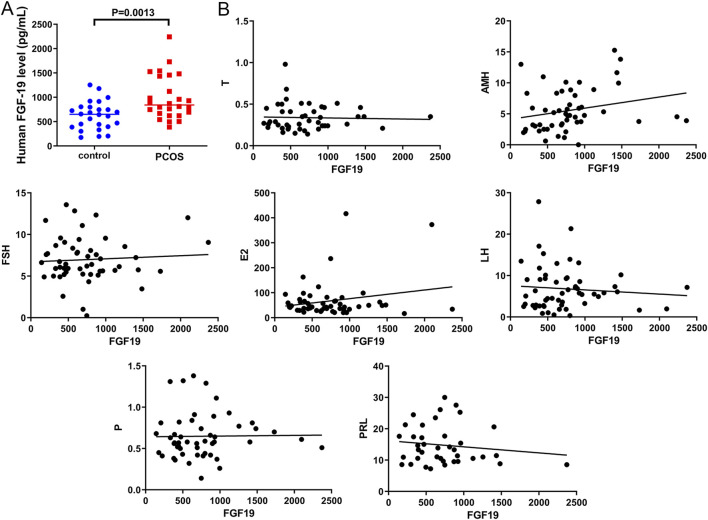
FGF19 expression in clinical samples and its correlation with polycystic ovarian syndrome (PCOS)-related indices **(A)** FGF19 expression in clinical PCOS samples. **(B)** The correlation between FGF19 expression and PCOS-related indices, including follicle-stimulating hormone (FSH), luteinizing hormone (LH), estradiol (E2), prolactin (PRL), progesterone (P), testosterone (T), and anti-Müllerian hormone (AMH).

To determine FGF19 levels in PCOS and the correlation between FGF19 levels and PCOS-related indices, blood samples were collected from PCOS patients. We found that patients with PCOS exhibited significantly higher FGF19 levels than healthy controls (*P* = 0.0013, Figure 1A). We further analyzed the correlation between FGF19 and PCOS-related indicators. No significant correlation was observed between FGF19 and FSH, LH, E2, PRL, AMH, progesterone, or testosterone levels (*P* > 0.05; Figure 1B). However, FGF19 levels were positively correlated with AMH, E2, and FSH levels and negatively correlated with LH and PRL levels (Figure 1B). These hormonal correlations suggest a potential interplay between FGF19 signaling and endocrine axes relevant to PCOS pathogenesis, which might influence both metabolic and proliferative phenotypes.

### 3.2 Identification of differential expressed proteins and functional analysis

**FIGURE 2:**
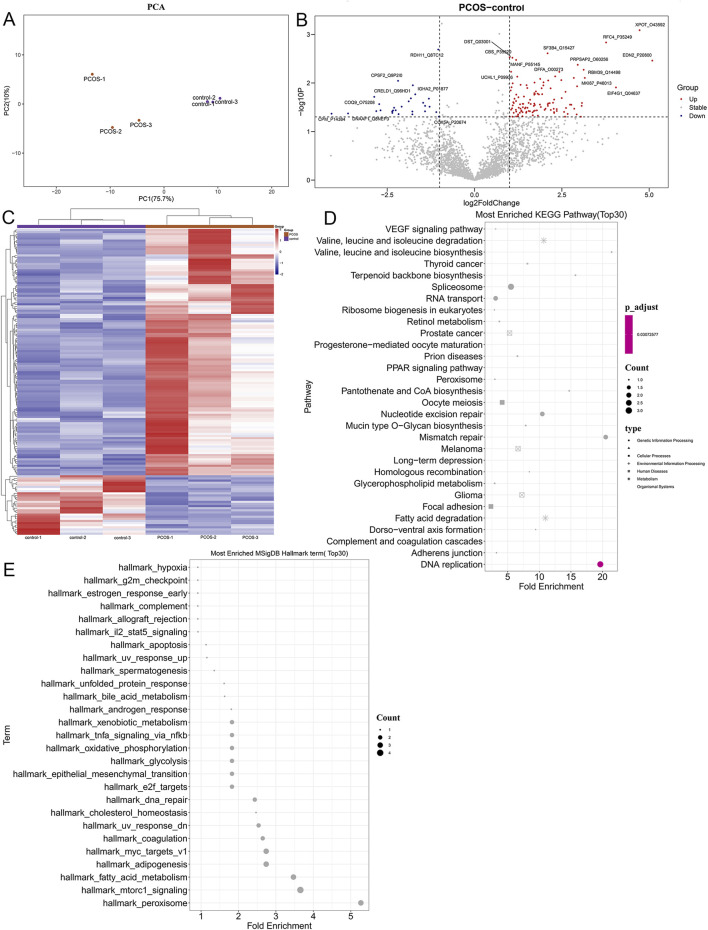
Identification of differentially expressed proteins and functional analysis. **(A)** PCA analysis of all samples. **(B)** The volcano plot of the identified differential expressed proteins with the thresholds of fold change ≥2.0, fold change ≤0.5, and p-value <0.05. **(C)** Heatmap of differentially expressed proteins (DEPs). **(D)** Kyoto Encyclopedia of Genes and Genomes (KEGG) analysis of identified DEPs. **(E)** Hallmark pathways of DEPs identified using Gene Set Enrichment Analysis (GSEA).

To further explore the molecular pathogenesis of PCOS, follicular fluid samples from PCOS patients and controls were used for proteomic analysis. Principal component analysis (PCA) showed that the samples in the PCOS and control groups were clearly separated (Figure 2A), indicating that all samples could be used for follow-up analyses. In total, 144 DEPs were identified, including 116 upregulated (XPOT, RFC4, EDN2, SF3B4, and PRPSAP2) and 28 downregulated DEPs (RDH11, CPSF2, IGHA2, CPM, and COQ9) (Figure 2B). The heatmap of the identified DEPs showed that the identified DEPs revealed a clear separation between control and PCOS samples (Figure 2C).

After that, the identified DEPs were subjected to functional analyses, containing KEGG pathways and hallmark pathways. As shown in Figure 2D, the identified DEPs were significantly enriched in “terpenoid backbone biosynthesis,” “VEGF signaling pathway,” “focal adhesion,” “PPAR signaling pathway,” “pantothenate and CoA biosynthesis,” “fatty acid degradation,” as well as “complement and coagulation cascades.” Furthermore, the identified DEPs were also involved in pathways of “bile acid metabolism” (related DEP: PRDX5), “hypoxia,” “oxidative phosphorylation,” “xenobiotic metabolism,” “IL-2-STAT5 signaling,” “apoptosis,” “TNF-α signaling via NF-κB,” “cholesterol homeostasis,” “epithelial mesenchymal transition (EMT),” as well as “mTORC1 signaling” (Figure 2E).

### 3.3 Construction of a PCOS mouse model, and related molecular pathogenesis

**FIGURE 3:**
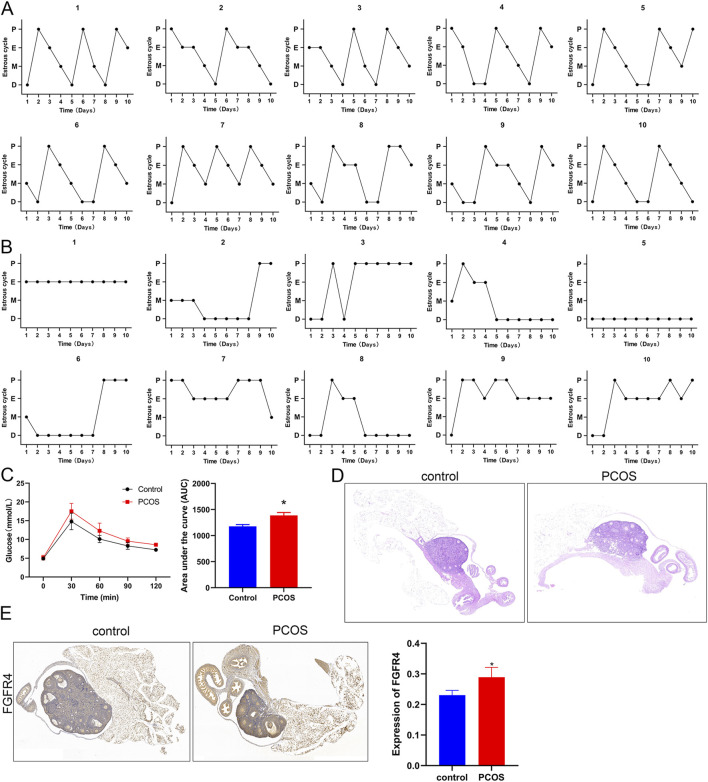
Construction of a PCOS mouse model and related indices **(A)** Estrous cycle of control mice for 10 consecutive days. **(B)** The estrous cycle of PCOS mice for 10 consecutive days. **(C)** Glucose levels and the area under the curve for control and PCOS mice. **(D)** Hematoxylin and eosin (HE) staining of mouse ovarian tissues at ×200 magnification. **(E)** Expression of FGFR4 in mice as determined by immunohistochemistry at ×200 magnification. *P < 0.05, vs. control.

In female mice, sexual maturation is accompanied by distinct estrous cycles under hormonal regulation, reflecting the ovulatory and endocrine functions of the ovaries. After model induction, daily vaginal smears were performed for 10 consecutive days to compare the estrous cycle patterns between the control and PCOS model groups. We observed that all control mice underwent phases of proestrus, estrus, metestrus, and diestrus (Figure 3A). However, PCOS mice exhibited disrupted estrous cyclicity, with some mice persistently remaining in estrus, while others were in diestrus, demonstrating significant heterogeneity in cycle patterns compared to controls (Figure 3B). Subsequently, the OGTT experiment showed that the glucose level in PCOS mice was evidently higher than that in control mice (*P* < 0.05, Figure 3C), suggesting impaired glucose tolerance in PCOS mice.

Histological examination of ovarian morphology was performed using HE staining. Control mice exhibited normal ovarian architecture with well-organized follicular development at all stages (primordial, primary, secondary, and antral follicles) and intact corpus luteum formation. The follicular structures were clearly demarcated, with distinct cellular morphologies (Figure 3D). In contrast, PCOS mice displayed multiple enlarged antral follicles characterized by sparse granulosa cells that were peripherally displaced, forming large follicular antrums (Figure 3D). Moreover, FGFR4 levels were significantly higher in mice with PCOS than in healthy animals, as demonstrated by IHC staining (Figure 3E).

**FIGURE 4:**
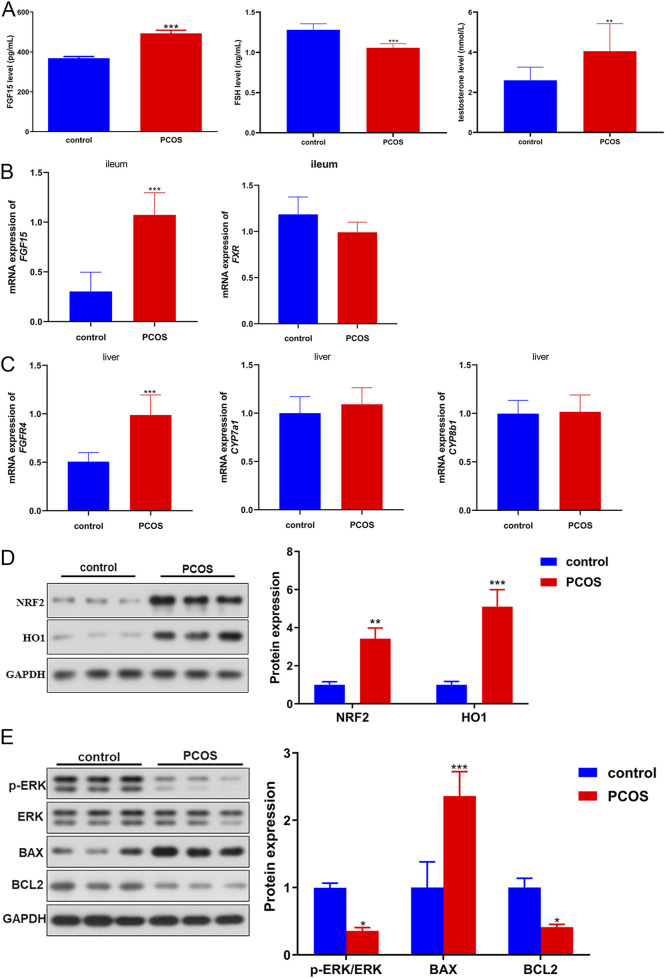
The Molecular pathogenesis of PCOS in a PCOS mouse model. **(A)** Levels of FGF15 (FGF19), FSH, and testosterone in mouse blood samples. **(B)** mRNA expression of *FGF15* and *FXR* in the ileal tissues of mice was determined using real-time quantitative PCR (RT-qPCR). **(C)** mRNA expression of *FGFR4*, *CYP7a1* and *CYP8b1* in the liver tissues of mice determined by RT-qPCR. **(D)** Protein expression of NRF2 and HO1 in mice, as determined by Western blotting. **(E)** Protein expression of p-ERK/ERK, BAX, and BCL2 in mice, as determined by Western blotting. *P < 0.05, **P < 0.01, ***P < 0.001, vs. control.

Further, the associated markers were measured using ELISA, RT-qPCR as well as Western blot. Compared to control mice, the levels of FGF15 and testosterone were significantly (*P* < 0.05) higher in the blood of PCOS mice, whereas the FSH level was significantly lower (Figure 4A). The ileum and liver tissues were collected for RT-qPCR. In the ileum tissues, a significant elevation in *FGF15* mRNA was observed in PCOS mice *versus* controls (*P* < 0.05), whereas *FXR* mRNA levels were comparable between the groups (*P* > 0.05; Figure 4B). Liver tissue analysis revealed significantly elevated *FGFR4* mRNA levels in PCOS mice compared to controls (*P* < 0.05); however, *CYP7a1* and *CYP8b1* mRNA levels were not significantly different between the groups (*P* > 0.05; Figure 4C). Furthermore, oxidative stress-related proteins (NRF2 and HO1), ERK pathway-related proteins, and apoptosis-related proteins (BAX and BCL2) were examined using Western blotting. NRF2 and HO1 expression was markedly (*P* < 0.05) elevated in PCOS mice compared to that in control mice (Figure 4D). Quantitative analysis revealed a significant increase in BAX and decrease in BCL2 expression in PCOS mice relative to controls (*P* < 0.05, Figure 4E). Additionally, compared with control mice, the level of p-ERK/ERK was markedly reduced in PCOS mice (*P* < 0.05; Figure 4E). Concurrent observations of impaired glucose tolerance, altered bile acid metabolism proteins, and hormonal disturbances suggest systemic metabolic-endocrine crosstalk in PCOS pathogenesis.

### 3.4 FGF19 regulates PCOS progression via ERK pathway *in vitro*

**FIGURE 5:**
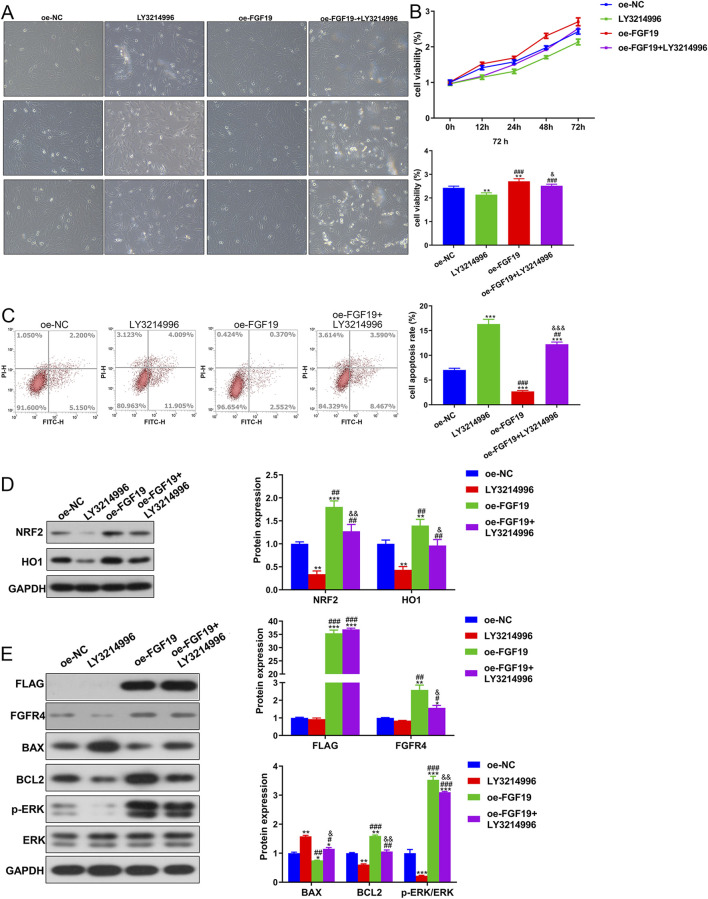
FGF19 regulates PCOS progression via the ERK pathway *in vitro*. **(A)** Changes in the cell survival state using a bright-field experiment at a magnification of ×200. **(B)** Viability of KGN cells subjected to different treatments using the cell counting kit-8. **(C)** Apoptosis of cells after different treatments was assessed using flow cytometry with the Annexin V-FITC/PI cell apoptosis assay kit. **(D)** Protein expression of NRF2 and HO1 in KGN cells subjected to different treatments as determined by Western blotting. **(E)** Protein expression levels of FLAG, FGFR4, p-ERK/ERK, BAX, and BCL2 in KGN cells subjected to different treatments as determined by Western blotting. The final DMSO concentration was 0.1%. *P < 0.05, **P < 0.01, ***P < 0.001, vs. oe-NC. ^#^ *P* < 0.05, ^##^ *P* < 0.01, ^###^ *P* < 0.001, vs. LY3214996. ^&^ *P* < 0.05, ^&&^ *P* < 0.01, ^&&&^ *P* < 0.001, vs. oe-FGF19.

Due to the high expression of FGF19 in PCOS, we performed *in vitro* experiments to elucidate the role of FGF19 in PCOS progression. Images of the survival of KGN cells treated with LY3214996 (an ERK inhibitor) or oe-FGF19 are shown in Figure 5A. Cell viability was quantified, and we observed that with an increase in culture time, the viability of KGN cells in different groups increased gradually. After 72 h of culture, KGN viability was significantly reduced by LY3214996 treatment, whereas it was significantly enhanced by transfection of oe-FGF19 compared with the oe-NC group (Figure 5B). However, compared with the FGF19 overexpressed KGN cells, LY3214996 treatment markedly decreased cell viability (Figure 5B). FGF19 overexpression significantly (*P* < 0.05) reduced the apoptosis of KGN cells; however, LY3214996 treatment markedly (*P* < 0.05) enhanced apoptosis caused by FGF19 overexpression (Figure 5C).

Western blotting revealed that NRF2 and HO1 protein expression was downregulated in the LY3214996-treated KGN cells, whereas it was markedly upregulated in the FGF19 over-expressed KGN cells compared to the non-NC-transfected KGN cells (Figure 5D). Relative to the FGF19 over-expressed KGN cells (*P* < 0.05), LY3214996 treatment markedly downregulated their expression (Figure 5D). Additionally, relative to oe-NC-transfected KGN cells, FLAG and FGFR were upregulated after FGF19 overexpression (*P* < 0.05); however, LY3214996 treatment had no significant effect on their expression relative to the oe-FGF19 group (*P* > 0.05, Figure 5E). BAX expression was significantly downregulated after FGF19 overexpression (*P* > 0.05), but LY3214996 treatment markedly upregulated its expression caused by FGF19 overexpression (*P* < 0.05; Figure 5E). Nonetheless, the expression trends of BCL2 and p-ERK/ERK were reciprocally related to the BAX levels in all treatment groups. (Figure 5E).

### 3.5 The interaction of FGF19 and NRF2 in PCOS induced by high oxidative stress

**FIGURE 6:**
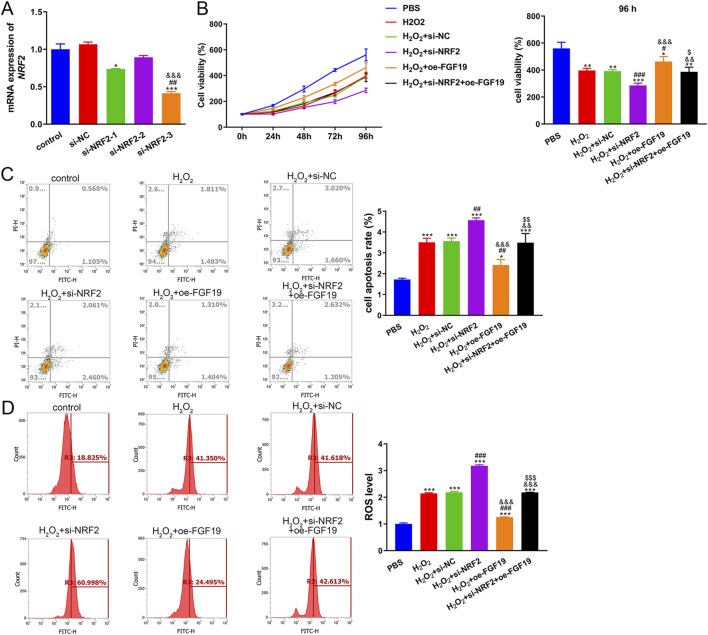
Interaction between FGF19 and NRF2 in high-oxidative stress-induced PCOS **(A)** Transfection efficiency was assessed by measuring the mRNA expression of *NRF2*. *P < 0.05, ***P < 0.001, vs. control. ^##^ *P* < 0.01, vs. si-NRF2-1. ^&&&^ *P* < 0.001, vs. si-NRF2-2. **(B)** Viability of KGN cells under high oxidative stress with different treatments using cell counting kit-8. **(C)** Apoptosis of KGN cells under high oxidative stress with different treatments using flow cytometry with an Annexin V-FITC/PI cell apoptosis assay kit. **(D)** ROS levels in KGN cells under high oxidative stress were evaluated by flow cytometry. *P < 0.05, **P < 0.01, ***P < 0.001, vs. PBS. ^#^ *P* < 0.05, ^##^ *P* < 0.01, ^###^ *P* < 0.001, vs. H_2_O_2_. ^&^ *P* < 0.05, ^&&^ *P* < 0.01, ^&&&^ *P* < 0.001, vs. H_2_O_2_+si-NFR2. ^$^ *P* < 0.05, ^$$^ *P* < 0.01, ^$$$^ *P* < 0.001, vs. H_2_O_2_+oe-FGF19.

To further explore oxidative stress in PCOS, KGN cells were treated with H_2_O_2_ and transfected with si-NRF2 or oe-FGF19. NRF2 expression remained unchanged between the control and si-NC groups (*P* > 0.05) but was significantly restrained by both si-NRF2-1 and si-NRF2-3 transfection, with si-NRF2-3 demonstrating superior knockdown efficiency (Figure 6A). Therefore, si-NRF2-3 was selected to construct KGN cells with *NRF2* silencing for further study. The viability of KGN cells after different treatments was examined using the CCK-8 assay. The viability of KGN cells in the different groups increased with increasing culture time (Figure 6B). Relative to the control cells, H_2_O_2_ induction evidently decreased cell viability (*P* < 0.05). NRF2 knockdown significantly reduced cell viability, whereas FGF19 overexpression markedly (*P* < 0.05) increased cell viability stimulated by H_2_O_2_ (Figure 6B). The apoptotic rate of KGN cells inversely correlated with cell viability across the treatment groups (Figure 6C). Furthermore, we measured ROS levels in different KGN cells under high oxidative stress conditions. Relative to the control conditions, H_2_O_2_-treated KGN cells exhibited significantly higher ROS production (*P* < 0.05). NRF2 knockdown further increased the ROS levels induced by H_2_O_2_ (*P* < 0.05), whereas FGF19 overexpression had the opposite effect (Figure 6D).

**FIGURE 7:**
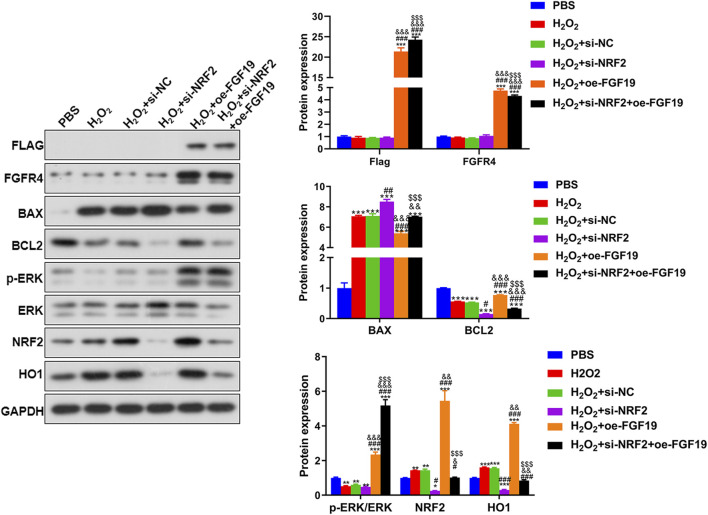
The protein expression of the related proteins in the KGN cells under high oxidative stress assessed by Western blot. Protein expression levels of FLAG, FGFR4, p-ERK/ERK, BAX, BCL2, NRF2, and HO1 in KGN cells after different treatments. *P < 0.05, **P < 0.01, ***P < 0.001, vs. PBS. ^#^ *P* < 0.05, ^##^ *P* < 0.01, ^###^ *P* < 0.001, vs. H_2_O_2_. ^&^ *P* < 0.05, ^&&^ *P* < 0.01, ^&&&^ *P* < 0.001, vs. H_2_O_2_+si-NFR2. ^$^ *P* < 0.05, ^$$^ *P* < 0.01, ^$$$^ *P* < 0.001, vs. H_2_O_2_+oe-FGF19.

Finally, Western blotting was performed to examine the levels of FLAG, FGFR4, BAX, BCL2, NRF2, HO1 and p-ERK/ERK in KGN cells treated with different methods under high oxidative stress. For FLAG-FGF19 and FGFR4, their protein expression showed no significant changes (*P* > 0.05) among the PBS, H_2_O_2_, H_2_O_2_+si-NC, and H_2_O_2_+si-NRF2 groups, whereas FGF19 overexpression significantly upregulated their expression relative to the control KGN. In the cells with NRF2 knockdown and FGF19 overexpression, FLAG-FGF19 was evidently upregulated while FGFR4 was significantly downregulated compared to the FGF19 overexpressed KGN (*P* < 0.05, Figure 7). Based on the apoptosis-related proteins, H_2_O_2_ administration significantly enhanced BAX and decreased BCL2 levels (*P* < 0.05), and NRF2 knockdown potentiated these effects, further elevating BAX and reducing BCL2 levels beyond the H_2_O_2_-induced changes (*P* < 0.05; Figure 7). However, compared to the H_2_O_2_+si-NRF2 group, FGF19 overexpression downregulated BAX expression and upregulated BCL2 expression (Figure 7). The expression of NRF2 and HO1 was significantly higher after H_2_O_2_ administration compared to the control KGN, and NRF2 silencing markedly decreased their expression caused by H_2_O_2_ (*P* < 0.05); however, FGF19 overexpression had the opposite effect on NRF2 knockdown (Figure 7). In addition, the level of p-ERK/ERK was lower in the H_2_O_2_ group than in the control KGN cells (*P* < 0.05), and NRF2 knockdown did not significantly alter the p-ERK/ERK levels caused by H_2_O_2_ (*P* > 0.05, Figure 7). Nonetheless, FGF19 overexpression significantly elevated p-ERK/ERK levels under high oxidative stress relative to H_2_O_2_-induced KGN (*P* < 0.05; Figure 7).

## 4 Discussion

PCOS is a multifaceted endocrine disorder that not only disrupts the female reproductive system, leading to fertility impairment, but also adversely affects the metabolic and cardiovascular systems, thereby increasing long-term health risks and exerting detrimental psychological consequences (Kulkarni et al., 2023). Since the first report published more than 2 decades ago, emerging evidence has consistently demonstrated that patients with PCOS exhibit elevated oxidative stress coupled with diminished antioxidant capacity (Sabuncu et al., 2001). FGF19, an endocrine hormone, is increasingly recognized to be involved in the pathogenesis and progression of PCOS (Bednarska and Siejka, 2017; Cheng et al., 2021). In the current study, increased FGF19 levels were first found in PCOS, which is in line with a previous study (Cheng et al., 2021). The correlation analysis between FGF19 and PCOS-related indices was then determined. Although no significant correlations were observed between FGF19 and individual PCOS-related indices (e.g., FSH, LH, and testosterone), a positive correlation with AMH (a marker of ovarian reserve) and E2 and a negative correlation with LH (a driver of androgen excess) suggest the potential involvement of FGF19 in follicular development and hormonal regulation. Consistent with previous reports that AMH levels are elevated in PCOS due to increased follicle numbers (Furat et al., 2018), our findings imply that FGF19 may contribute to aberrant folliculogenesis by modulating AMH-related pathways. Additionally, the lack of a strong correlation with testosterone contrasts with studies linking FGF19 to metabolic dysfunction in PCOS (Cheng et al., 2021), indicating that the role of FGF19 may be more prominent in oxidative stress and ERK-mediated cellular processes rather than in direct androgen regulation.

Further to explore the molecular pathogenesis of PCOS, proteomics was performed on follicular fluid samples to identify 144 DEPs between the healthy individuals and PCOS patients, which enriched in TNF-α signaling via NF-κB, VEGF signaling pathway, PPAR signaling pathway, IL-2-STAT5 signaling, epithelial mesenchymal transition (EMT), bile acid metabolism, mTORC1 signaling, as well as oxidative phosphorylation. VEGF-mediated ovarian angiogenesis is essential for normal follicular development and corpus luteum formation, and elevated VEGF expression has been consistently observed in patients with PCOS (Morsi et al., 2022). A previous comprehensive *in silico* analysis revealed that VEGF signaling is involved in the development of follicles in the ovaries of women with PCOS (Patil et al., 2022). PPARs are ligand-activated transcription factors that modulate energy homeostasis and play key roles in glucose and fatty acid metabolism (Wagner and Wagner, 2020). Daghestani et al. (2022) analyzed granulosa cells from PCOS patients with or without a history of ovarian hyperstimulation syndrome by transcriptome, and observed significant alterations in the JAK/STAT, PPAR, and NF-κB signaling pathways. Furthermore, IL-2-STAT5 signaling has been identified as a crucial immune regulatory mechanism that primarily governs T cell proliferation and differentiation, which are closely related to PCOS (Li S. et al., 2024). The mTORC1 signaling pathway regulates metabolism and cell growth in response to nutrient levels; mTORC1 expression is upregulated in PCOS, whereas it is downregulated by naringenin and morin in PCOS rats (Yi et al., 2022). It has been reported that PCOS patients displayed the increased abundance of local inflammatory factor proteins in endometrial cells; as well as TNF-α could influence PCOS progression by activating the p-NF-κBp65 signaling pathway (Ha et al., 2021). Bile acids not only impair nutrient and vitamin absorption but also serve as critical signaling molecules that regulate glucose and lipid metabolism. Notably, patients with PCOS exhibit significantly elevated total bile acid levels compared with healthy controls (Yu et al., 2023). EMT can promote ovarian tissue fibrosis associated with PCOS, affecting ovarian function and hormonal balance (Liu et al., 2025). Furthermore, oxidative phosphorylation is reported to play important roles in oocyte development, insulin resistance, and ovulation disorders and is closely related to the occurrence and development of PCOS (Lu et al., 2022). Taken together, we speculated that the functions of EMT, bile acid metabolism, and oxidative phosphorylation; as well as the signaling pathways of IL-2-STAT5, VEGF, PPAR, mTORC1, and TNF-α signaling via NF-κB, enriched by the identified 144 DEPs (such as XPOT, RFC4, EDN2, SF3B4, and PRPSAP2, RDH11, CPSF2, IGHA2, CPM, and COQ9), may have crucial effects on PCOS evolvement. However, the precise roles of the identified DEPs and their enriched functions in PCOS need to be further verified.

Follicular fluid, a dynamic microenvironment surrounding developing oocytes, contains various cell types shed from ovarian tissues, including granulosa cells (the primary somatic cells supporting folliculogenesis), cumulus cells, oocyte-corona radiata complexes, and occasional immune cells (e.g., macrophages) (Ni et al., 2022; Basuino and Silveira, 2016). These cells are critical for regulating follicle maturation, hormone synthesis, and oxidative balance, which are key processes that are disrupted in PCOS. Intracellular signaling pathways identified by proteomics (e.g., mTORC1-, ERK-, and NRF2-mediated oxidative stress responses) are predominantly associated with granulosa cells, which play a central role in follicular development and are known to exhibit aberrant function in PCOS (Liao et al., 2022). For instance, granulosa cells (KGN) express FGFR4 (as shown in our IHC and *in vitro* data) and are highly sensitive to oxidative stress, making them a plausible source of DEPs linked to oxidative phosphorylation and EMT observed in our follicular fluid analysis. Additionally, cumulus cells, which interact closely with oocytes, may contribute to the DEPs involved in VEGF signaling (critical for angiogenesis during follicle growth) (Abumaghaid et al., 2022) and PPAR pathways (regulating lipid metabolism in ovarian cells) (Cadenas et al., 2025). Thus, the intracellular pathways enriched in our proteomic analysis likely reflect functional changes in granulosa and cumulus cells, which is consistent with our *in vitro* findings using KGN cells (a human granulosa cell line) and supports the relevance of follicular fluid DEPs in PCOS pathogenesis.

Recently, accumulating data have indicated that PCOS is associated with gut dysbiosis (Elkafas et al., 2022), which is characterized by a significant increase in *Bacteroides* populations (Sun Y. et al., 2023) and altered bile acid profiles with increased levels of deoxycholic acid, chenodeoxycholic acid, and lithocholic acid (Yang X. et al., 2021). These changes are correlated with insulin and testosterone levels, suggesting that bile acid metabolic homeostasis directly or indirectly influences PCOS pathogenesis (Yu et al., 2023; Yang X. et al., 2021). Our proteomic analysis identified PRDX5 as a key DEP enriched in bile acid metabolism in the PCOS follicular fluid. PRDX5, a member of the peroxiredoxin family with peroxidase and chaperone activities, plays dual roles in redox regulation and bile acid metabolism (Chen et al., 2020). Its downregulation in PCOS follicular fluid may reflect both oxidative stress adaptation and altered bile acid signaling. Emerging evidence suggests that bile acids can influence ovarian function through FXR (Chiang and Ferrell, 2020; Režen et al., 2022) and that PRDX5-mediated regulation of bile acid metabolism may contribute to the dysregulated follicular microenvironment in PCOS. This finding is consistent with recent reports on altered bile acid profiles in patients with PCOS (Yu et al., 2023), suggesting a potential novel axis connecting hepatic metabolism, oxidative stress, and ovarian dysfunction. The specific mechanisms by which alterations in PRDX5 expression and bile acid metabolism influence the pathogenesis of PCOS warrant further investigation.

In addition, following meal ingestion, intestinal bile acids activate FXR, a nuclear receptor central to bile acid signaling (Bozadjieva-Kramer et al., 2024). Interaction between bile acids and FXR initiates FGF19/FGF15 expression FGF19/FGF15 is a hormone transmitted through enterohepatic circulation and its production is regulated by FXR activity in the ileum and various bile acid species at different concentrations in the gut (Katafuchi and Makishima, 2022). Mechanistically, FGF19/FGF15 can bind to and activate the hepatic FGFR4-β-klotho receptor complex, subsequently regulating markers linked to bile acid homeostasis, cholesterol metabolism, as well as lipid metabolism, further modulating expression of apoptosis-associated genes (BAX, as well as BCL2) (Katafuchi and Makishima, 2022). CYP7A1 and CYP8B1 encode rate-limiting enzymes involved in bile acid biosynthesis (Rizzolo et al., 2021). In this study, the expression of these factors was investigated in a mouse model of PCOS. Glucose tolerance was impaired in PCOS mice, and FGF15 and FGFR4 were significantly upregulated. However, no significant alterations in hepatic FXR, CYP7A1, or CYP8B1 expression were observed in PCOS mice relative to controls. Moreover, we found that NRF2 and HO1 were upregulated, whereas p-ERK/ERK levels were decreased in PCOS mice. Therefore, these findings suggest the importance of FGF15/FGF19, FGFR4, the ERK pathway, and oxidative stress in PCOS.

Our study also revealed elevated levels of NRF2 and HO1 in PCOS mice compared to controls (Figure 4D). This finding aligns with the well-documented role of oxidative stress in the pathogenesis of PCOS. Under chronic oxidative stress, NRF2, a master regulator of antioxidant responses, is activated to counteract excessive ROS accumulation (Glorieux et al., 2024). HO1, a downstream target of NRF2, exerts cytoprotective effects by degrading pro-oxidant heme into antioxidant molecules, such as biliverdin and carbon monoxide (O'Rourke et al., 2024). In PCOS, hyperandrogenism and insulin resistance disrupt redox homeostasis, leading to sustained oxidative stress (Lőrincz et al., 2024). The upregulation of NRF2 and HO1 likely represents a compensatory mechanism to mitigate oxidative damage in ovarian tissues. However, despite this adaptive response, persistent oxidative insult in PCOS may overwhelm the antioxidant system and contribute to follicular dysfunction and metabolic disturbances. Further studies are warranted to determine whether NRF2/HO1 activation in PCOS is protective or maladaptive in the long-term.

Furthermore, we investigated the specific roles of the FGF15 and ERK pathways in PCOS *in vitro* and observed that FGF19 overexpression facilitated the viability of KGN cells while inhibiting their apoptosis; however, LY3214996 (an ERK inhibitor) reversed the action of FGF19 overexpression in KGN cells. Qi et al. (2023) illustrated that APOC3 is upregulated in KGN cells, and silencing APOC3 reduces the viability of KGN cells and induces their apoptosis, thereby promoting PCOS progression. Another study showed that ACTN1 is highly expressed in ovarian cancer (OC) cells and tissues, and its knockdown can reduce the invasion, proliferation, and migration of OC cells while promoting apoptosis, whereas LY3214996 (an ERK inhibitor) partially reversed the actions of ACTN1 knockdown (Sun et al., 2025). Furthermore, we observed that FGF19 overexpression upregulated FGFR4, NRF2, HO1, BCL2, and p-ERK/ERK and downregulated BAX, while LY3214996 had the opposite effects of FGF19 overexpression. FGFR4 is downstream of FGF19, and ELF4 upregulation induced by FGF19 signaling drives colorectal cancer metastasis through FGFR4 transcriptional activation (Chen et al., 2023). NRF2 as well as HO1 are closely associated with oxidative stress. A previous study showed that remote ischemic regulation attenuates oxidative stress and neuroinflammation in MCAO-reperfusion mice via NRF2/HO-1 pathway activation, resulting in improved neurological outcomes (Sun YY. et al., 2023). BCL2 is a pro-apoptotic protein, whereas BAX is an anti-apoptotic protein that jointly regulate apoptosis (Kharrazi et al., 2024). Fu et al. (2022) reported that *Ligularia fischeri* root extract improved ulcerative colitis in mice by activating the BCL2/BAX signaling pathway to reduce inflammation and protect intestinal epithelial cells and intestinal epithelial barriers. Together with our results, these reports suggest that FGF19 may participate in PCOS development by regulating FGFR4, cell apoptosis-related proteins (BAX and BCL2), oxidative stress-related proteins (NRF2 and HO1), and ERK pathways.

Substantial evidence has established that oxidative stress is a crucial pathogenic factor in multiple metabolic disorders, particularly type 2 diabetes mellitus, obesity, PCOS, and cardiovascular disease (Dubey et al., 2021). The critical threshold for oxidative stress is reached when the rate of oxidant formation exceeds the cellular reducing potential (Teleanu et al., 2022). Free radicals interact with micromolecules and macromolecules (e.g., lipids, proteins, and DNA) through oxidative modifications, resulting in structural and functional damage (Hajam et al., 2022). Oxidative stress causes DNA and protein damage. In PCOS, oxidative stress drives key pathological features, including impaired insulin signaling, elevated androgen levels, and low-grade inflammation (Dubey et al., 2021). Women with PCOS exhibit abnormal levels of oxidative stress biomarkers (e.g., glutathione, malondialdehyde, and superoxide dismutase), along with an imbalance in total antioxidant capacity, which further impairs cellular repair mechanisms and enhances susceptibility to oxidative damage (Uçkan et al., 2022). Under physiological conditions, moderate ROS levels are essential for critical reproductive processes including oocyte maturation, folliculogenesis, and embryonic development (Mohammadi, 2019). However, excessive ROS generation disrupts mitochondrial DNA integrity, triggers apoptotic pathways, and may contribute to adverse reproductive outcomes such as infertility and pregnancy loss (Dabravolski et al., 2021). Understanding the interplay between oxidative stress and PCOS progression is crucial for developing targeted therapeutic strategies. Our study showed that H_2_O_2_ treatment evidently decreased the viability of KGN cells while increasing apoptosis and ROS levels. NRF2 knockdown further aggravated the effect of H_2_O_2_, whereas FGF19 overexpression improved the action of H_2_O_2_. Furthermore, we also observed that H_2_O_2_ stimulation upregulated BAX, NRF2, and HO1, while decreasing the levels of BCL2 and p-ERK/ERK; NRF2 knockdown further upregulated BAX and downregulated BCL2 and p-ERK/ERK. However, FGF19 overexpression had the opposite effects on NRF2 knockdown. A previous investigation of Sun et al. (2023c) illustrated that kisspeptin was reduced in the ovarian granulosa cells of PCOS rats, and its overexpression enhanced proliferation, inhibited the apoptosis of KGN cells, and suppressed ROS production by activating PI3K/AKT/ERK signaling. Based on our results, we speculate that FGF19 may regulate PCOS progression through the mediation of NRF2 and the oxidative stress response.

Our findings revealed a potential interconnected network linking hormonal dysregulation, cellular proliferation, and metabolic alterations in PCOS. The observed correlations between FGF19 and PCOS-related hormonal indices (Figure 1) suggest crosstalk between FGF19 signaling and the hypothalamic-pituitary-ovarian axis. Specifically, a positive association with AMH and a negative association with LH may reflect FGF19 modulation of follicular development and androgen production (Ramanjaneya et al., 2020; Cheng et al., 2021). These hormonal changes likely contribute to the proliferative phenotype observed in KGN cells because sex steroids are known to regulate granulosa cell growth and survival (Luo et al., 2021). Simultaneously, altered bile acid metabolism (via PRDX5) and impaired glucose tolerance in PCOS mice suggest that metabolic dysfunction may result from and exacerbate these hormonal imbalances. Bile acids can function as signaling molecules that influence glucose homeostasis through FXR activation (Xiang et al., 2023), and the hyperinsulinemia characteristic of PCOS may further disrupt bile acid metabolism (Yang Y. et al., 2021). This creates a potentially vicious cycle in which hormonal dysregulation impairs metabolic function, which in turn worsens endocrine disturbances. The FGF19-FGFR4 axis may serve as a key modulator at this intersection, given its established roles in both bile acid and glucose metabolism (Wu et al., 2011) and our demonstration of its effects on granulosa cell proliferation. Collectively, these data suggest that metabolic reprogramming of the glucose and bile acid pathways in PCOS creates a microenvironment that favors excessive granulosa cell proliferation and abnormal hormone secretion, contributing to the pathophysiology of polycystic ovaries.

## 5 Conclusion

FGF19 is highly expressed in patients with PCOS. Proteomics analysis showed EMT, bile acid metabolism, oxidative phosphorylation, and the signaling pathways of IL-2-STAT5, VEGF, PPAR, mTORC1, as well as TNF-α signaling via NF-κB, enriched by the identified 144 DEPs, may play important roles in PCOS evolvement. In addition, FGF19 may be involved in PCOS occurrence and development of by regulating the FGFR4-ERK-NRF2 pathway. These findings advance our understanding of the etiology of PCOS and identify FGF19 and ERK pathway modulation as viable therapeutic strategies.

## Data Availability

The original contributions presented in the study are included in the article/supplementary material, further inquiries can be directed to the corresponding authors.
